# Association between *ADCY9* Gene Polymorphisms and Ritodrine Treatment Outcomes in Patients with Preterm Labor

**DOI:** 10.3390/pharmaceutics13101653

**Published:** 2021-10-10

**Authors:** Nari Lee, Ha-Young Yoon, Jin-Young Park, Young-Ju Kim, Han-Sung Hwang, Jeong Yee, Hye-Sun Gwak

**Affiliations:** 1 College of Pharmacy and Graduate School of Pharmaceutical Sciences, Ewha Womans University, Seoul 03760, Korea; leenarisme@gmail.com (N.L.); hayoungdymphnayoon@gmail.com (H.-Y.Y.); kellyjeans87@naver.com (J.-Y.P.); 2 Department of Obstetrics and Gynecology, Ewha Womans University School of Medicine, Seoul 07985, Korea; kkyj@ewha.ac.kr; 3 Department of Obstetrics and Gynecology, Konkuk University Medical Center, Konkuk University School of Medicine, Seoul 05030, Korea; hwanghs@kuh.ac.kr

**Keywords:** adenylyl cyclase 9 gene, adverse drug events, preterm labor, ritodrine, single nucleotide polymorphism, time to delivery

## Abstract

The purpose of this study was to investigate the genetic effects of *ADCY9* on ritodrine responses in patients with preterm labor. Five single nucleotide polymorphisms (SNPs) of the *ADYC9* gene in 163 patients in preterm labor were genotyped: rs879619, rs2601796, rs2531988, rs2531995, and rs2230739. Additionally, rs598961 of the *PDE4B* gene and rs1042719 of the *ADRB2* gene were included for analysis. Patients with CC genotype of *ADCY9* rs879619 had a 2.0-fold (95% confidence interval [CI]: 1.3, 3.2) higher hazard of time to delivery than T allele carriers. Patients with combined genotypes of CC in *ADCY9* rs879619, AA in *PDE4B* rs598961, and GC, CC in *ADRB2* rs1042719 showed a greater hazard of time to delivery than patients with other combinations (adjusted hazard ratio [AHR] 3.2; 95% CI: 1.7, 6.3), whereas patients carrying the C allele of *ADCY9* rs2531995, G allele of *PDE4B* rs598961, and GG genotype of *ADRB2* rs1042719 had a lower hazard of time to delivery than patients carrying other genotypes (AHR 0.4; 95% CI: 0.2, 0.7). Regarding ritodrine-induced adverse drug events (ADEs), height less than 160 cm and CC genotype of *ADCY9* rs2531995 showed a greater risk of ADEs. The results of our study suggest that *ADCY9* polymorphisms could affect the efficacy and safety of β_2_-adrenergic agonists.

## 1. Introduction

Preterm birth is a delivery occurring before 37 weeks of gestation and its global incidence is estimated at 15 million annually [1]. As preterm birth is the leading cause of neonatal morbidity and mortality, appropriate tocolytic agents (including β_2_-adrenergic agonists) are recommended for the treatment of preterm labor.

Ritodrine binds to β_2_-adrenergic receptors (ADRB2) on uterine smooth muscle, resulting in increased levels of intracellular cyclic adenosine-3′,5′-monophosphate (cAMP), which leads to relaxation of the uterine smooth muscle [2,3,4]. In previous studies, genetic polymorphisms of *ADRB2* and phosphodiesterase 4B (*PDE4B*) were considered important factors for ritodrine treatment response by regulating the intracellular concentration of cAMP [5,6].

Adenylate cyclase (ADCY), which is stimulated by G protein-coupled receptors (such as ADRB2), catalyzes the conversion of ATP to cAMP and controls smooth muscle contractility. Among the ADCY family, ADCY9 is classified into a separate category which is only activated by the Gs alpha subunit [7]. The *ADCY9* gene is encoded on chromosome 16. Several studies have shown that *ADCY9* polymorphisms are associated with responses of airway smooth muscle to β_2_-adrenergic agents [8,9,10]. Although *ADCY9* polymorphisms could also have effects on the uterine smooth muscle [7], no studies have been conducted in the treatment of preterm labor. Therefore, this study aimed to investigate the potential role of *ADCY9* gene polymorphisms that could alter ritodrine efficacy and ritodrine-induced adverse drug events (ADEs) in pregnant women with preterm labor.

## 2. Methods

### 2.1. Patients

This was a prospective study conducted between January 2010 and December 2014 at Ewha Womans University Mokdong Hospital. We enrolled patients aged ≥ 18 years who received ritodrine treatment for preterm labor (i.e., uterine contractions of more than 3 in 10 min and cervical change) with an intact membrane at gestational age 20 to 36 weeks and provided written informed consent. Patients were excluded if (1) they received ritodrine treatment for McDonald procedure; (2) they had pre-eclampsia, fetal distress, severe oligohydramnios, placenta abruption, placenta previa, or spontaneous premature membrane rupture; (3) their continuation of pregnancy would be detrimental; (4) they had cardiovascular disease, hyperthyroidism, or asthma, which could preclude the detection of outcomes; or (5) there were no blood samples. This study was performed in accordance with ethical standards of the Declaration of Helsinki and approved by the Institutional Review Board (IRB) of Ewha Womans University Mokdong Hospital (No. 217-1-26, 6 January 2010). Informed consent was obtained from all patients before enrollment.

### 2.2. Drug Administration

Ritodrine (Lavopa^®^; JW Pharmaceutical, Seoul, Korea) by intravenous infusion started at the rate of 0.05 mg/min and increased by 0.05 mg/min every 10 min until uterine quiescence was achieved. After that, maintenance therapy was given for 12–48 h at a dose of 0.05 mg/min.

### 2.3. Outcomes and Data Collection

We reviewed medical records and collected data, including maternal age, height, weight, body mass index, gestational age at drug administration, modified Bishop score, multiple pregnancy, and delivery information.

The primary outcome was time to delivery, which was defined as the time interval from initiation of ritodrine therapy to fetal delivery, and the secondary outcome was ritodrine-induced ADEs. ADEs were defined as tachycardia over 100 beats/min, palpitation, dyspnea, shortness of breath, or pulmonary edema requiring drug discontinuation or dose reduction.

### 2.4. Selection of Single Nucleotide Polymorphisms (SNPs)

SNPs of *ADCY9* were selected based on other studies [8,9,10]. Genetic information (e.g., rsID, chromosomal position, reference/alternate allele, and function) was obtained from the University of California Santa Cruz genome browser [11] and Asian minor allele frequency (MAF) and linkage disequilibrium (LD) data of each SNP were collected from Haploreg v4.1 [12]. Using the tagger function of the Haploview v4.2 [13], *ADCY9* SNPs were assorted. SNPs were considered redundant in case of strong LD (r^2^ ≥ 0.8). In addition, *PDE4B* and *ADRB2* SNPs, which were reported in previous studies as affecting ritodrine efficacy and ADEs, were included for analysis [5,6].

### 2.5. Genotyping Methods

Genomic DNA was extracted from EDTA-treated whole blood using QIAamp DNA Blood Mini Kits (QIAGEN, Hilden, Germany). *PDE4B* rs598961 (c.232-1587 G > A) and the following *ADCY9* SNPs were genotyped by a single-base primer extension assay using SNaPShot Multiplex kit (Applied Biosystems, Foster City, CA, USA): rs879619 (c.*730 C > T), rs2601796 (c.1694-30417 C > T), rs2531988 (c.2679 + 700 T > G), rs2531995 (c.*2309 C > T), and rs2230739 (c.13 T > C). In addition, TaqMan genotyping assay was used for *ADRB2* rs1042719 (c.1053 G > C).

### 2.6. Statistical Analyses

Time to delivery data were summarized by the Kaplan–Meier method, and log-rank test was used to determine statistical differences between two groups. To compare patients with and without ADEs, chi-squared and Fisher’s exact tests were carried out. Each SNP was tested in both dominant and recessive models, and the most appropriate model was selected by considering both effect size and statistical significance. As ADCY9, PDE4B, and ADRB2 shared the cAMP signaling pathway in uterine relaxation, we also investigated the combined genetic effects of *ADCY9/PDE4B/ADRB2* on time to delivery by grouping genotypes with similar trends and comparing them.

A Cox proportional hazards regression model and logistic regression model were used to identify the independent factors for time to delivery and ritodrine-induced ADEs, respectively. Multivariable analysis with backward elimination was performed with variables of *p* < 0.05 in univariate analysis in addition to known factors (age and *ADRB2* rs1042719 [5]).

The Hosmer–Lemeshow goodness-of-fit test was used to evaluate the model fitting. An analysis of the area under the receiver operating curve (AUROC) was conducted to assess the ability of risk factors to predict ritodrine-induced ADEs. All statistical analysis was performed using SPSS 20.0 software (IBM Corp., Armonk, NY, USA). A *p* < 0.05 was considered statistically significant.

### 2.7. In Silico Analyses

The Polymorphism in microRNAs and their TargetSites (PolymiRTs database; University of Tennessee Health Sciences Center, Memphis, TN, USA) was used to predict the functional impact of polymorphisms in the 3-prime untranslated region (3’-UTR) [14]. Using information about 3’-UTR sequence, this bioinformatics tool provides potential SNPs that could create, destroy, or modulate an miRNA-binding site. The context+ score, which incorporated site-type, 3’ pairing, local AU, position, target site abundance, and seed-pairing stability, was used to evaluate the miRNA binding to the 3′-UTR sequence [15]. The score with a more negative value indicates the more likelihood of target site disruption or new site creation [14].

## 3. Results

A total of 236 patients were enrolled, and 163 were eligible for study. Seventy-three patients were excluded due to the following reasons: ritodrine treatment for McDonald procedure (*n* = 10), severe cases requiring labor before arriving at the hospital (*n* = 15), underlying cardiovascular diseases (*n* = 10), and lack of samples (*n* = 38).

Mean maternal age and height were 30.9 ± 3.5 years and 161.3 ± 4.4 cm, respectively. Mean weight and BMI were 62.4 ± 4.4 kg and 24.0 ± 4.7 kg/m^2^, respectively. Gestational age at drug therapy ≥ 32 weeks (*p* < 0.001) and modified Bishop score ≥ 3 (*p* = 0.001) were statistically significant factors for time to delivery (Table 1). Except for two cases of miscarriage, the percentage of spontaneous preterm birth was 47%. Mean gestational age at delivery was 36.2 ± 3.8 weeks.

The genotypes and MAF of studied SNPs of the *ADCY9* gene are shown in Table 2. All genotype distributions were consistent with the Hardy–Weinberg equilibrium. In univariate analysis, rs879619 (C > T) and rs2531995 (C > T) of *ADCY9* and rs1042719 (G > C) of *ADRB2* were significantly associated with time to delivery. T allele carriers of rs879619 had a longer time to delivery than patients with CC genotype (*p* = 0.018). For rs2531995, patients carrying TT genotype showed a shorter time to delivery than C allele carriers (*p* = 0.039). C allele carriers of rs1042719 in the *ADRB2* gene had a shorter time to delivery than those with GG genotype (*p* = 0.012). The effects of combined genotypes of the *ADCY9*, *PDE4B* and *ADRB2* SNPs on time to delivery were also analyzed. Patients with the CC genotype of *ADCY9* rs879619, the AA genotype of *PDE4B* rs598961, and C allele of *ADRB2* rs1042719 had a shorter median time to delivery (*p* = 0.004), whereas patients carrying the C allele of *ADCY9* rs2531995, G allele of *PDE4B* rs598961, and GG genotype of *ADRB2* had a longer median time to delivery (*p* < 0.001) compared to patients with other genotypes, respectively.

Table 3 shows results from multivariable regression analysis after adjusting for factors with *p* < 0.05, besides age and *ADRB2* rs1042719 (Model I). Patients carrying CC genotype of *ADCY9* rs879619 had a greater hazard of time to delivery than patients with T allele (adjusted hazard ratio [AHR] 2.0; 95% confidence interval [CI]: 1.3, 3.2). On the contrary, age and rs2531995 failed to remain in the final model. Model II included combined genotypes of *ADCY9* rs879619, *PDE4B* rs598961, and *ADRB2* rs1042719 instead of *ADCY9* rs879619 of Model I. Patients with the combined genotypes of CC in *ADCY9* rs879619, AA in *PDE4B* rs598961, and C allele in *ADRB2* rs1042719 showed a greater hazard of time to delivery than patients with other combinations (AHR 3.2; 95% CI: 1.7, 6.3). Model III was constructed with the combined genotype of *ADCY9* rs2531995, *PDE4B* rs598961, and *ADRB2* rs1042719 instead of *ADCY9* rs2531995 of Model I. Patients carrying the C allele of *ADCY9* rs879619, G allele of *PDE4B* rs598961, and GG genotype of *ADRB2* rs1042719 had a lesser hazard of time to delivery than patients carrying other genotypes (AHR 0.4; 95% CI: 0.2, 0.7). Gestational age at drug therapy (≥32 weeks) and modified Bishop score (≥3) were significant factors regardless of the constructed models.

For ritodrine-induced ADEs, height (*p* = 0.009) and rs2531995 (C > T) of the *ADCY9* gene (*p* = 0.013) showed a significant association with ADEs (Table 4). After adjusting for factors with *p* < 0.05, besides age and rs1042719 of the *ADRB2* gene, height < 160 cm, and CC genotype of *ADCY9* rs2531995 showed a greater risk of ADEs (adjusted odds ratio [AOR] 2.5, 95% CI: 1.2, 5.2; and AOR 2.2, 95% CI: 1.1, 4.2, respectively) (Table 5). The Hosmer-Lemeshow test for ADEs, which included height and rs2531995, showed a good fit (χ^2^ = 0.174, *p* = 0.917). The AUROC was 0.660 (95% CI: 0.567, 0.753).

*ADCY9* rs879619 and rs2531995 were located in the 3’-UTR region. The results from PolymiRTs showed that *ADCY9* rs2531995 ( C > T) created new binding sites for has-miR-632, has-miR-4288, and has-miR-346. Context+ score differences were –0.126, –0.108, and –0.069, respectively. *ADCY9* rs879619 (C > T) created a new binding site for has-miR-3186-Sp, and its context+ score difference was –0.126.

## 4. Discussion

This study revealed that *ADCY9* rs879619, gestational age at drug therapy, and modified Bishop score were significant factors for time to delivery after ritodrine administration. *ADCY9* rs2531995 and height were significantly associated with ritodrine-induced adverse events.

T allele carriers of *ADCY9* rs879619 showed a significantly longer median time to delivery. Although 3’-UTR regions, in which *ADCY9* rs8796195 is located, are a non-coding part of mRNA, they are known to regulate gene expression through degradation, translation, and localization of mRNAs [16]. Therefore, it was speculated that *ADCY9* rs879619 could change expression of the *ADCY9* gene and its protein. Consistent with our findings, this polymorphism was also found to be associated with percent change in maximum mid-expiratory flow in response to bronchodilators in a previous study investigating the pharmacogenetic effects of *ADCY9* in patients with asthma [9].

Ile772Met, also known as *ADCY9* rs2230739, is the most commonly studied SNP of the *ADCY9* gene. In a transfected cell line, Met772-expressing cells had a significantly lower level of ß-agonist-stimulated ADCY activity [8,17]. However, Korean asthmatics carrying Met772 showed a better percent change in forced expiratory volume after 8 weeks’ administration of a bronchodilator in a clinical trial [9]. Although patients with CC genotype of *ADCY9* rs2230739 had almost half the median time to delivery than that of T allele carriers, this difference failed to reach statistical significance in our study. This was possibly due to the small sample size.

Ritodrine binds to ADRB2 and activates ADCY to catalyze the formation of cAMP from ATP; however, PDE terminates the signaling of smooth muscle relaxation by degrading cAMP to 5’-AMP [3,7]. Therefore, the *ADCY9* gene could interact with the *PDE4B* and *ADRB2* genes in prolonging time to delivery by regulating the intracellular cAMP concentration. After combining genotypes of *ADCY9* rs879619, *PDE4B* rs598961, and *ADRB2* rs1042719, AHR for time to delivery increased to 3.2, relative to a value of 2.0 for *ADCY9* rs879619 alone.

A previous study evaluating the interaction between *ADCY9* gene polymorphisms and asthma in Brazilian children [10], and another trial using genome-wide association studies in obesity [18], indicated that *ADCY9* rs2531995 was responsible for *ADCY9* expression and intracellular cAMP production. In our study, *ADCY9* rs2531995 alone failed to achieve statistical significance in multivariable analysis; however, it showed a significant effect after being combined with *PDE4B* rs598961 and *ADRB2* rs1042719. *ADCY9* rs2531995, located in the 3’-UTR region, was also a significant factor in multivariable logistic regression of ritodrine-induced ADEs.

Through our bioinformatics analysis, we found that the variant alleles (T alleles) of rs879619 and rs2531995 created new binding sites for miRNA, indicating that both SNPs were potential regulatory elements for post-transcription *ADCY9* gene expression. Importantly, miRNA regulates the stability and translation of mRNA by binding to specific regions in the 3’-UTR [19].

Regarding patient characteristics, older gestational age at drug therapy, and higher modified Bishop score were significant risk factors for shorter time to delivery. This can be attributed to the fact that modified Bishop score consists of the degree of cervical dilation and effacement, which are important risk factors for preterm birth [20]. Unexpectedly, lower height was associated with ritodrine-induced ADEs. Further research is required to examine this association.

The limitations of this study are related to its single-center design with a small sample size. With 163 patients, the power to detect a clinically significant decrease of 35% in time to delivery [21] was calculated to 74% and 58% for SNPs with MAF of 20% and 10%, respectively. Especially for rs2601796, whose observed MAF was around 10%, it might be more difficult to detect differences than for other SNPs. To afford enough statistical power and validate our hypothesis, further multicenter studies with large sample sizes are needed. The second limitation was a lack of considering the family history of adverse pregnancy outcomes and developmental abnormalities, which could affect preterm labor. However, there is major clinical value in that this is the first study to evaluate a potential role for *ADCY9* in pregnant women with preterm labor. Moreover, the results can be applied to respiratory diseases such as asthma and chronic obstructive pulmonary disease.

This study suggests that *ADCY9* polymorphisms can affect the efficacy and safety of β_2_-adrenergic agonists. The results may be appropriate for creating individualized treatment plans in pregnant women with preterm labor.

## Figures and Tables

**Table 1:** Effects of demographic characteristics on time to delivery.

Characteristic	Number of Patients	Time to Delivery Median (95% CI)	*p* Value
Age (years)			
<30	59	1168.6 (764.3–1572.8)	0.492
≥30	104	1040.0 (670.5–1409.5)	
Height (cm)			
<160	53	1027.2 (580.6–1473.7)	0.419
≥160	110	1168.6 (873.7–1463.4)	
Weight (kg)			
<60	66	978.6 (476.1–1481.0)	0.983
≥60	97	1230.6 (964.5–1496.7)	
BMI (kg/m^2^)			
<25	112	1247.8 (940.7–1554.9)	0.934
≥25	51	1040.0 (656.0–1424.0)	
Gestational age at drug therapy (weeks)			
<32	111	1408.1 (1172.8–1643.5)	<0.001
≥32	52	548.0 (272.0–824.0)	
Modified Bishop score ^a^			
<3	109	1247.8 (1112.9–1382.8)	0.001
≥3	19	156.9 (25.5–288.3)	
Multiple pregnancy ^b^			0.207
Single	125	1072.6 (800.2–1345.1)	
Multiple	18	631.1 (147.2–1114.9)	

^a^ Modified Bishop score is the sum of dilatation score and effacement score. Dilatation score: 0, <l cm; 1, 1 to 3 cm; 2, 3 to 5 cm; 3, ≥5 cm. Effacement score: 0, 0% to 40%; 1, 40% to 60%; 2, 60% to 80%; 3, ≥80%. 35 missing data for modified Bishop score. ^b^ 20 missing data for multiple pregnancy.

**Table 2:** Effects of grouped genotypes on time to delivery.

Gene Polymorphism	Minor Allele Frequency (%)	Grouped Genotype	Number of Patients	Time to Delivery Median (95% CI)	*p* Value
*ADCY9*					
rs879619 (C > T) ^a^	27.4	CC	82	811.6 (532.9–1090.4)	0.018
		CT, TT	79	1300.8 (1187.1–1414.5)	
rs2601796 (C > T) ^a^	7.6	CC, CT	24	978.6 (495.4–1461.7)	0.650
		TT	137	1168.6 (874.4–1462.7)	
rs2531988 (T > G)	28.6	TT, GT	83	1275.4 (974.3–1576.4)	0.565
		GG	80	934.7 (679.5–1190.0)	
rs2531995 (C > T)	35.0	CC, CT	147	1190.7 (961.0–1420.4)	0.039
		TT	16	613.9 (0–1237.0)	
rs2230739 (T > C) ^b^	38.2	TT, CT	142	1168.6 (940.2–1396.9)	0.340
		CC	20	613.9 (42.4–1185.5)	
*PDE4B*					
rs598961 (G > A) ^c^	37.1	GG, GA	89	1298.9 (1162.2–1435.7)	0.102
		AA	58	828.6 (553.3–1103.9)	
*ADRB2*					
rs1042719 (G > C) ^b^	44.5	GG	53	1338.5 (950.1–1727.0)	0.012
		GC,CC	109	978.6 (680.5–1276.6)	
*ADCY9/PDE4B/ADRB2*					
rs879619/rs598961/rs1042719 ^d^	N/A	CC/AA/ GC,CC	20	568.6 (0–1261.9)	0.004
		Others	125	1274.8 (1077.3–1432.2)	
rs2531995/rs598961/rs1042719 ^c^	N/A	CC,CT/GG,GA/GG	25	1875.5 (1084.2–2666.8)	<0.001
		Others	122	978.6 (738.4–1218.7)	

N/A: not available. ^a^ 2 missing data for rs879619 and rs2601796, ^b^ 1 missing data for rs2230739 and rs1042719, ^b^ 2 missing data for rs2601796, ^c^ 16 missing data for rs598961 and rs2531995/rs598961/ rs1042719, ^d^ 18 missing data for rs879619/rs598961/rs1042719.

**Table 3:** Multivariable analysis of time to delivery.

Factor	Model I	Model II	Model III
	Adjusted HR (95% CI)	Adjusted HR (95% CI)	Adjusted HR (95% CI)
Gestational age at drug therapy (≥32 weeks)	4.0 (2.4–6.9) ***	4.7 (2.7–8.3) ***	4.1 (2.3–7.3) ***
Modified Bishop score (≥ 3)	2.1 (1.2–3.9) *	2.2 (1.2–4.1) *	2.3 (1.2–4.3) *
*ADRB2* rs1042719 (GC,CC)	1.6 (1.0–2.6)		
*ADCY9* rs879619 (CC)	2.0 (1.3–3.2) **		2.1 (1.3–3.3) **
*ADCY9* rs879619/*PDE4B* rs598961 /ADRB2 rs1042719(CC/AA/GC,CC)		3.2 (1.7–6.3) **	
*ADCY9* rs2531995/*PDE4B* rs598961 /*ADRB2* rs1042719 (CC,CT/GG,GA/GG)			0.4 (0.2–0.7) **

* *p* < 0.05, ** *p* < 0.01, *** *p* < 0.001. All factors, of which *p*-value was < 0.05 from univariate analysis in addition to age were included in Model I. Model II included combined genotype of *ADCY9/PDE4B/ADRB2* rs879619/rs598961/rs1042719 instead of *ADCY9* rs879619 of Model I. Model III included combined genotype of *ADCY9/PDE4B/ADRB2* rs2531995/rs598961/rs1042719 instead of *ADCY9* rs2531995 of Model I.

**Table 4:** Effects of demographic characteristics and grouped genotypes in *ADCY9* on adverse drug events.

Characteristic	Number of Patients (%)	Adverse Drug Events		*p* Value
		Yes (n = 46)	No (n = 117)	
Age (years)				
<30	59 (36.2)	15 (32.6)	44 (37.6)	0.550
≥30	104 (63.8)	31 (67.4)	73 (62.4)	
Mean ± S.D	31.0 ± 3.7	31.0 ± 4.4	30.9 ± 3.5	0.880
Height (cm)				
<160	53 (32.5)	22 (47.8)	31 (26.5)	0.009
≥160	110 (67.5)	24 (52.2)	86 (73.5)	
Mean ± S.D	161.2 ± 4.5	160.3 ± 4.7	161.6 ± 4.4	0.083
Weight (kg)				
<60	66 (40.5)	24 (52.2)	42 (35.9)	0.057
≥60	97 (59.5)	22 (47.8)	75 (64.1)	
Mean ± S.D	62.5 ± 8.5	61.0 ± 8.2	63.0 ± 8.6	0.176
BMI (kg/m^2^)				
<25	112 (68.7)	33 (71.7)	79 (67.5)	0.601
≥25	51 (31.3)	13 (28.3)	38 (32.5)	
Mean ± S.D	24.0 ± 3.0	23.7 ± 2.8	24.1 ± 3.2	0.458
Gestational age at drug therapy(weeks)				
<32	111 (68.1)	33 (71.7)	78 (66.7)	0.532
≥32	52 (31.9)	13 (28.3)	39 (33.3)	
Mean ± S.D	29.7 ± 3.7	29.0 ± 3.9	29.9 ± 3.6	0.151
Modified Bishop score ^a^				
<3	109 (85.2)	32 (91.4)	77 (82.8)	0.221
≥3	19 (14.8)	3 (8.6)	16 (17.2)	
Multiple pregnancy ^b^				0.098
Single	125 (87.4)	35 (79.5)	90 (90.9)	
Multiple	18 (12.6)	9 (20.5)	9 (9.1)	
rs879619 (C > T) ^c^				
CC	82 (50.9)	22 (47.8)	60 (52.2)	0.618
CT, TT	79 (49.1)	24 (52.2)	55 (47.8)	
rs2601796 (C > T) ^c^				
CC, CT	24 (14.9)	7 (15.6)	17 (14.7)	0.886
TT	137 (85.1)	38 (84.4)	99 (85.3)	
rs2531988 (T > G)				
TT, GT	83 (50.9)	22 (47.8)	61 (52.1)	0.620
GG	80 (49.1)	24 (52.2)	56 (47.9)	
rs2531995 (C > T)				
CC	64 (39.3)	25 (54.3)	39 (33.3)	0.013
CT, TT	99 (60.7)	21 (45.7)	78 (66.7)	
rs2230739 (T > C) ^d^				
TT	66 (40.7)	21 (45.7)	45 (38.8)	0.423
CT, CC	96 (59.3)	25 (54.3)	71 (61.2)	
*PDE4B* rs598961 (G > A) ^e^				
GG, GA	89 (60.5)	20 (52.6)	69 (63.3)	0.246
AA	58 (39.5)	18 (47.4)	40 (36.7)	
*ADRB2* rs1042719 (G > C) ^d^				
GG, GC	125 (77.2)	37 (82.2)	88 (75.2)	0.341
CC	37 (22.8)	8 (17.8)	29 (24.8)	
*ADCY9/ADRB2* rs2531995/rs1042719 ^d^				
CC/GG,GC	16 (9.9)	8 (17.8)	8 (6.8)	0.073
Others	146 (90.1)	37 (82.2)	109 (93.2)	

^a^ 35 missing data for modified Bishop score, ^b^ 20 missing data for multiple pregnancy, ^c^ 2 missing data for rs879619 and rs2601796, ^d^ 1 missing data for rs2230739, *ADRB2* rs1042719, and rs2531995/rs1042719, ^e^ 16 missing data for *PDE4B* rs598961.

**Table 5:** Multivariable analysis of adverse drug events.

Factor	Unadjusted OR (95% CI)	Adjusted OR (95% CI)
Age (≥ 30)	1.2 (0.6–2.6)	
Height (< 160 cm)	2.5 (1.1–5.2) *	2.5 (1.2–5.2) *
*ADRB2* rs1042719 (GG,GC)	1.5 (0.6–3.6)	
*ADCY9* rs2531995 (CC)	2.4 (1.2–4.8) *	2.2 (1.1–4.2) *

* *p* < 0.05. All factors, of which *p*-value was < 0.05 from univariate analysis in addition to age and *ADRB2* rs1042719 were included.

## Data Availability

The data that support the findings of this study are available from the corresponding author upon reasonable request.
